# Recent Advancements and Challenges in Lignin Valorization: Green Routes towards Sustainable Bioproducts

**DOI:** 10.3390/molecules27186055

**Published:** 2022-09-16

**Authors:** Mati Ullah, Pengyang Liu, Shangxian Xie, Su Sun

**Affiliations:** 1 College of Urban Construction, Wuchang Shouyi University, Wuhan 430070, China; 2 Department of Biotechnology, College of Life Science and Technology, Huazhong University of Science and Technology, Wuhan 430074, China

**Keywords:** lignin, biocatalysis, valorization, green synthesis

## Abstract

The aromatic hetero-polymer lignin is industrially processed in the paper/pulp and lignocellulose biorefinery, acting as a major energy source. It has been proven to be a natural resource for useful bioproducts; however, its depolymerization and conversion into high-value-added chemicals is the major challenge due to the complicated structure and heterogeneity. Conversely, the various pre-treatments techniques and valorization strategies offers a potential solution for developing a biomass-based biorefinery. Thus, the current review focus on the new isolation techniques for lignin, various pre-treatment approaches and biocatalytic methods for the synthesis of sustainable value-added products. Meanwhile, the challenges and prospective for the green synthesis of various biomolecules via utilizing the complicated hetero-polymer lignin are also discussed.

## 1. Introduction

The most profuse and renewable energy source on the planet is lignocellulosic biomass derived from plants; it comprises the two carbohydrates polymers, cellulose and hemicellulose, with the phenolic polymer lignin. The lignocellulosic biomass provides an alternative basis to fossil fuels for second-generation biofuels and other biobased chemicals production; however, the lignin biomass delivers excessive recalcitrance [1,2]. Lignin is a non-carbohydrate aromatic hetero-polymer with rich content and a complex structure in nature, present in the walls of the vascular tissue of plants [3,4]. This aromatic polymer is believed to be the second-most-abundant renewable resource, accounting for up to 25% of the total land-based biomass. Lignin does not have a fixed composition in all plant species, as its composition varies in different plant species; variation even exists in different tissues of the same plant. Cork lignin is mostly acetylated on the γ-OH of the side-chain (forty-eight percent acetylation) over the G units, whereas the lignin from the phloem and the xylem are barely acetylated, and this occurs mainly on the S units [5,6,7]. 

**Figure 1:**
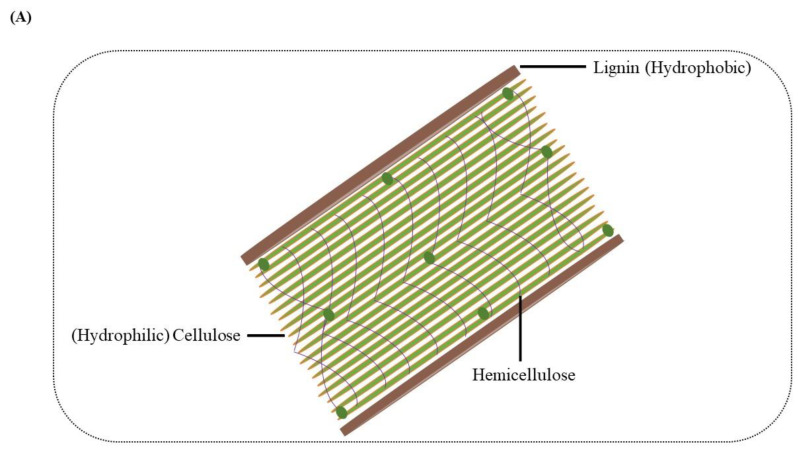

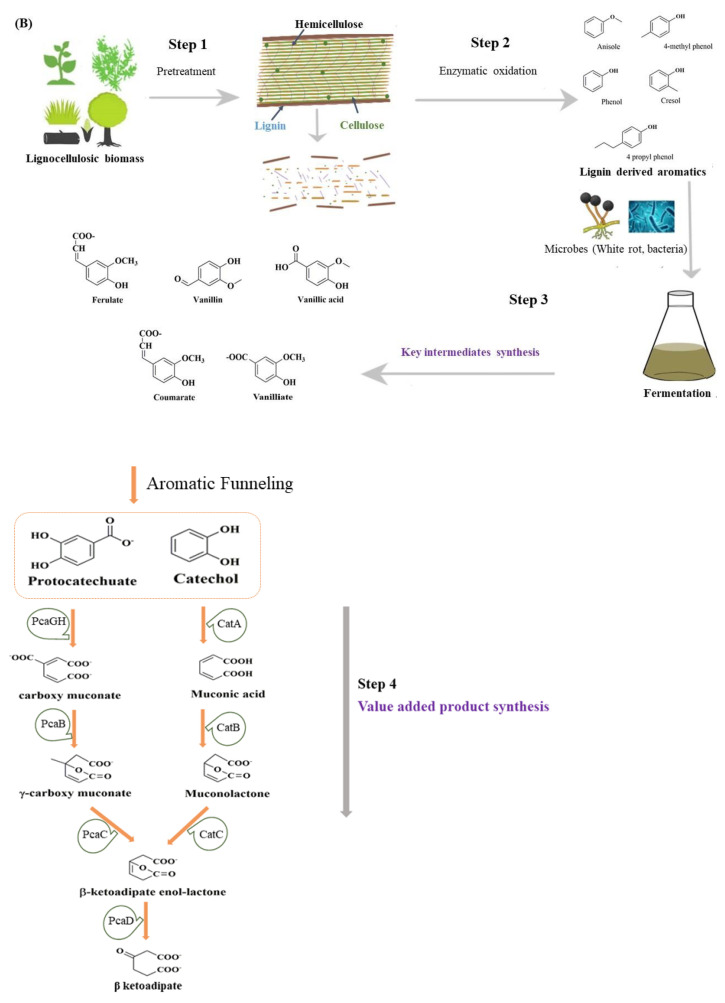

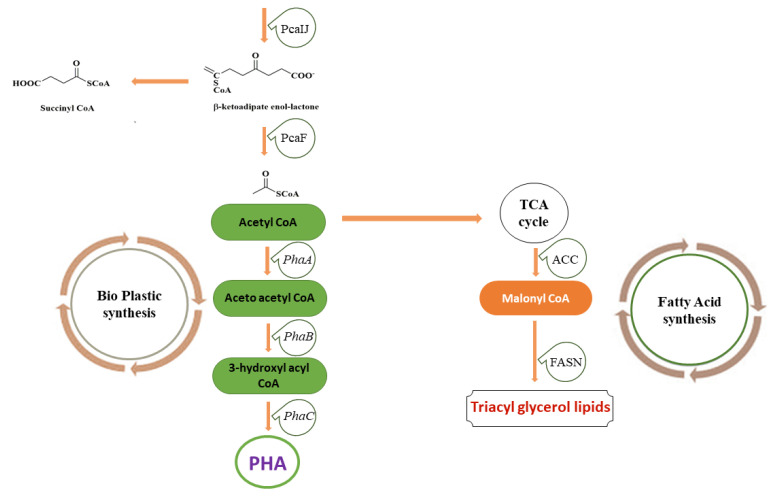
(**A**) Composition of the plant cell wall. Arrangement of hydrophobic lignin and hydrophilic cellulose along with other cell wall structure components. (**B**) Value-added intermediate products synthesis from lignin. In step 1, the pretreatment of the lignocellulosic biomass occurs, separating cellulose, hemicellulose and lignin. The enzyme depolymerization of the lignin takes place in step 2, generating certain lignin-derived aromatics. The aromatics are hydrolyzed in step 3 through the microbial action of lignin-degrading white rot or bacteria, leading to the formation of key intermediates, including protocatechuate and catechol. The protocatechuate and catechol on further conversion and entering the β-ketoadipate pathway lead to value-added products like triglycerides.

Lignin was considered a waste product in biorefinery during the production of biofuel from lignocellulosic biomass. After extraction, this polymer received attention and was found to have much potential as a natural reservoir of different chemicals and valuable products [8]. Thus, the release of lignin from conventional biomass refineries along with the pulp and paper industry has resulted in urgent requirements and also created the opportunity to increase the transformation of lignin into value-added products dramatically [9,10]. Many chemical and physical methods were explored to acquire the desired compounds from the lignin, but they all have certain limitations. Hence, for obtaining access to polysaccharides, the microbes have to break down or modify the lignin molecular structure [11,12]. The polysaccharide in the cell wall is hydrophilic, while the lignin is hydrophobic (Figure 1A); this combination of hydrophobic lignin and hydrophilic polysaccharides gives plants a significant advantage. The need for energy consumption for the non-specific breaking of these rich energy bonds is highly challenging; thus, the microbial degradation of lignin diverted the researchers’ attention and interest. For the better bioconversion of residual lignin-enriched refinery waste from upstream pretreatment, the most strategic process is to depolymerize macromolecular lignin into small molecular aromatic compounds for further conversion through microorganisms [13]. This conversion is similar to saccharification in cellulose; however, unlike the β-1,4-glucosidic linkage of cellulose, lignin contains diverse aromatic monomers and various types of chemical bonds or interunit linkages such as β-O-4, α-O-4/β-5, β–β, 4-O-5, and 5–5 [10]. 

Scientists are still working to find economical and reliable methods to degrade lignin for acquiring the desired breakdown products [14,15]. Several enzymes for lignin depolymerization have been discovered, along with the essential auxiliary enzymes involved in valuable compound synthesis. The white and brown-rot fungi contributing to this degradation produce extracellular peroxidase and laccase enzymes, along with the involvement of some peripheral enzymes [15,16,17]. Certain lignin-degrading bacteria are also considered significant for contributing to the bioconversion and biocatalysis of lignin through their enzymes [18,19]. 

The bioconversion of lignin mainly involves lignin pretreatment, the depolymerization of lignin into small molecular aromatic compounds, their degradation into central metabolites or key intermediates, and, lastly, the value-added product synthesis by microorganisms as represented in Figure 1B. The lignin depolymerization facilitates the conversion of low-molecular-weight lignin into monomers and oligomers, the degradation of monomers/oligomers into archetypal substrates, e.g., protocatechuate (PCA), the formation of acetyl-CoA from PCA through the β-KAP pathway, and the synthesis of lipid/PHA or other important molecules [20,21,22]. The current study focuses on the biodegradation and biodepolymerization of lignin and the challenges of meeting the bioconversion and valorization of lignin to value-added chemicals.

## 2. Lignin Availability and Structure

**Figure 2:**
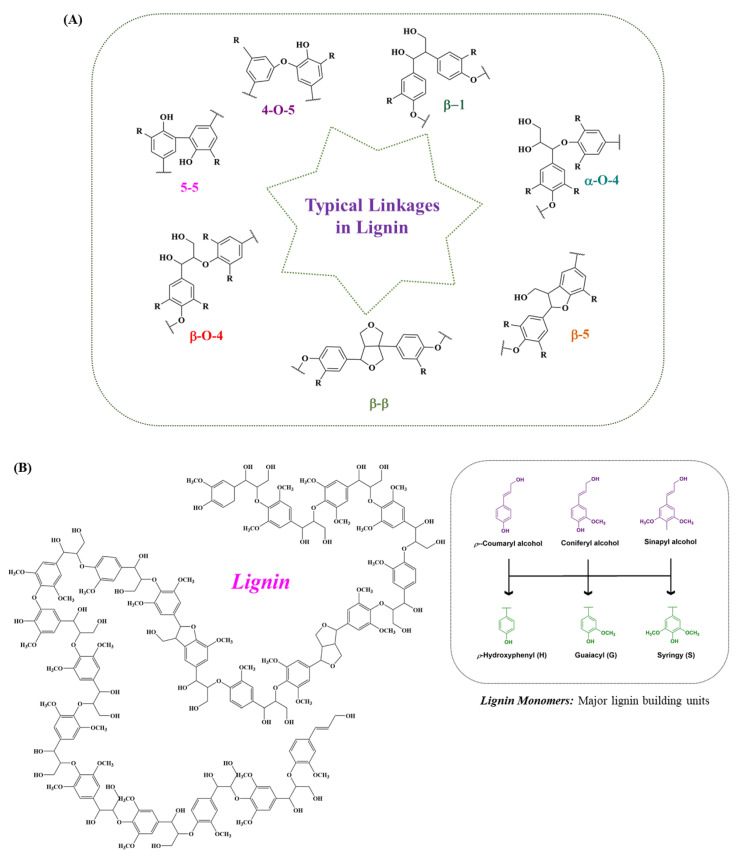
Typical linkages present in lignin. (**A**) The hetero-polymer lignin is naturally featured with a branched and cross-linked network of phenylpropane units. These lignin-forming units are conjugated by different linkages, such as β-O-4, α-O-4, β-1, β-5, 5-5, β-β, and 4-O-5. From the lignin structure, it is observed that the dominant inter-unit linkages present in the native lignin structure are the β-aryl ether (β-O-4). (**B**) Structure of lignin. The combination of different lignin monomers, mainly sinapyl alcohol, coniferyl alcohol and p-coumaryl alcohol, forms a stable lignin structure through energy-rich bonds.

Lignin is technically intertwined with cellulose and hemicellulose to form the main structure of plants, providing plants with strength and rigidity. The synthesis of lignin occurs via the free-radical-assisted enzymatic dehydrogenative polymerization of phenylpropanoid precursors, namely p-coumaryl alcohol (H), sinapyl alcohol (S) and coniferyl alcohol (G) [7,23] (Figure 2A). Thus, optically inactive amorphous heteropolymer (lignin) is naturally featured, with a branched and cross-linked network of phenylpropane units (C9). The ratio of lignin subunits differs among different plant species at different growth stages. The lignin monomers are conjugated by different bonds, such as β-O-4, β-5, β-1, 5-5, β-β, α-O-4 and 4-O-5. The β-aryl ethers (β-O-4) are the dominant inter-unit linkages in the native lignin structure, as shown in Figure 2B. The subunits are cross-linked with the polysaccharides present in the xylem and phloem tissue, contributing to recalcitrance by preventing microbial attract from infiltrating into cell walls [24,25]. Achievements have been made to effectively enhance lignocellulosic biomass conversion by increasing the syringl residues ratio. The lignocellulose conversion can also be enhanced by introducing more ester linkages via alternative monolignols expression [24,26]. The composition of depolymerized lignin varies significantly based on the source of the feedstock and how the feedstock is processed. Besides the biomass variations, the lignin isolation methods also have an influential role in defining the structure and nature of lignin. Like the two primary processes involved in the pulp and paper industry, separating lignin from carbohydrates includes kraft and sulfite pulping; thus, the lignin is termed kraft lignin and lignosulfonate [1,27,28]. Similarly, soda lignin involves treatment with soda or alkali, while the lignin formed due to a mixture of organic ethanol and water as solvents from lignocellulose is referred to as organosolv process lignin [29,30]. Additionally, the various other advanced isolation methods can lead to the formation of certain types of lignin, including milled wood lignin, cellulolytic enzyme lignin and enzymatic mild acidolysis lignin [31,32,33]. The types of lignin based either on the different feedstocks or pre-treatment methods along with their monomers’ molecular weights are given in Table 1, while the other advanced methods for lignin isolation, along with the average molecular weights of the extracted lignin, their advantages, and limitations are presented in Table 2. 

## 3. Overview on Advancements in Lignin Pretreatment and Valorization into Fine Chemicals

In the biorefinery process, a pretreatment step is usually applied to reduce the recalcitrance of lignin and increase solubilizing hemicellulose to expose the crystalline cellulose core to be hydrolyzed by cellulase enzymes for ethanol production. Most lignocellulosic biorefineries use thermochemical pretreatment steps coupled to enzymatic hydrolysis for deconstructing plant polysaccharides, hence yielding lignin-rich streams [44,45]. Lignin can be retained as soluble and fractionated before downstream carbohydrate conversion; it can also be kept as an insoluble residue after extracting most of the carbohydrates by hydrolysis or by pretreatment [46]. It is noteworthy that high-severity pretreatment hydrolysis or deconstruction approaches will chemically modify lignin. The lignin fractionation method alters the chemical bonds and functional groups of lignin, such as cleaving labile C-O linkage and reforming more recalcitrant C-C linkages, which will affect the reactivity and bioconversion efficiency of lignin [47,48,49]. 

**Figure 3:**
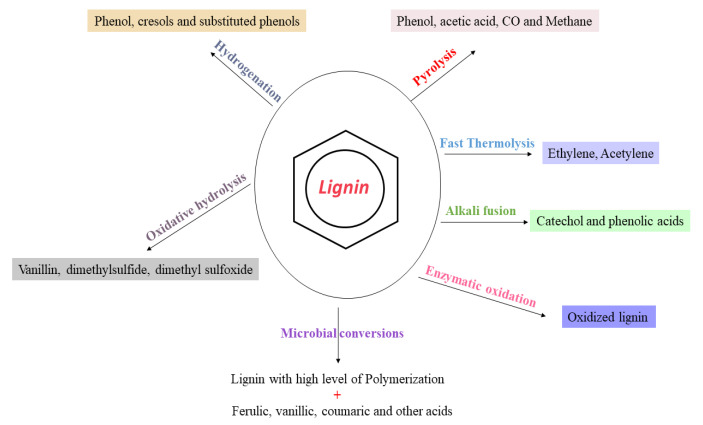
Value-added chemicals formed from lignin through various treatments.

**Figure 4:**
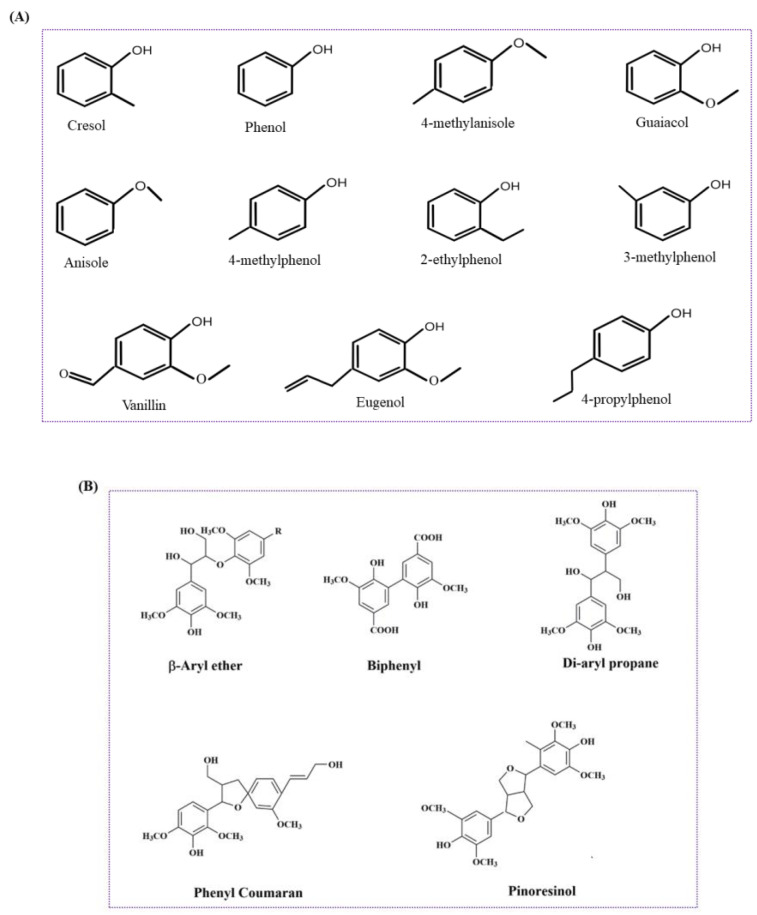
(**A**) Lignin-derived monomeric bio-oils and phenolic compounds. (**B**) Lignin-derived oligomers.

Several efforts have been rationalized to valorize lignin into fuels and valuable chemicals, as shown in Figure 3, which include catalytic pyrolysis, oxidation and hydrotreatment (hydrogenolysis and deoxygenation). Thermochemical treatment (e.g., pyrolysis) is the most-often considered method for rendering a series of heterogeneous mixtures of aromatic species or lignin fragments (i.e., C6–C22) [50,51]. It is reported that the pyrolysis of softwood kraft lignin generates a massive amount of heavy oil and char [52]. However, almost all of the thermochemical strategies result in an arsenal of several monomeric, oligomeric and polymeric compound mixtures, constituting an intricate composition of biooil [53] (Figure 4A,B). Simultaneously, for biofuel generation, an additional expensive and cumbersome hydrodeoxygenation step is required to avoid repolymerization and a self-condensation reaction. The partial hydrodeoxygenation product in a hydroprocessing step is then used to obtain the final desired biofuel [54,55]. Hydrotreatment is an approach with high selectivity, a high lignin conversion rate and the significant reduction of coke content. Shao et al. demonstrated the selective production of liquid hydrocarbons (C7–C9) via the direct hydrodeoxygenation of organosolv lignin over a potential porous catalyst Ru/Nb_2_O_5_ in water [56].

Xu and Liguori used formic acid as a hydrogen source, combined with Pt and Pd as catalysts, to convert lignin into relatively prominent phenolic species [57]. Transition-metal catalysts have been used for lignin hydrogenolysis and hydrodeoxygenation, since most of the catalysts, such as Ni-Mo and Co-Mo/Al_2_O_3_ are neither cheap, nor recyclable or robust. The various reactivities of lignin-derived compounds also result in poisons to metal catalysts in biomass-derived streams [58,59]. Several thermochemical or chemical catalytic approaches are carried out to establish potential lignin high-grade platforms; however, industrial utilization is always hindered by its cost, complex compositions, low quality, energy consumption and organic waste treatment [60,61]. Recently, some breakthroughs were achieved by Rahimi et al., who demonstrated a method for the depolymerization of oxidized lignin under a mild condition in aqueous formic acid combined with a metal catalyst that results in more than 60% (wt) aromatic monomer production [62].

Similarly, lignin-consolidated bioprocessing can also plays a prominent role in the valorization of lignin into valuable chemicals. One such example is the development of combinatorial pretreatment to fractionate lignin from corn stover that improved lignin reactivity and increased lipid production. In a previous study, Xie reported that choosing bmr mutants sorghum (sorghum bicolor) with *Cunninghamella echinulate* FR3 can convert biomass without chemical pretreatment. Similarly, the dilute acid pretreatment of biomass resulted in more weight loss during fungal fermentation than untreated biomass, which showed complete biomass utilization in a consolidated platform without chemical pretreatment [63]. Furthermore, *cis*,*cis*-muconate produced with engineered *P. putida* grown on a biomass-derived lignin-enriched stream demonstrated an integrated strategy towards lignin valorization, forming a vital product [64].

## 4. Physical, Chemical and Physicochemical Depolymerization

**Figure 5:**
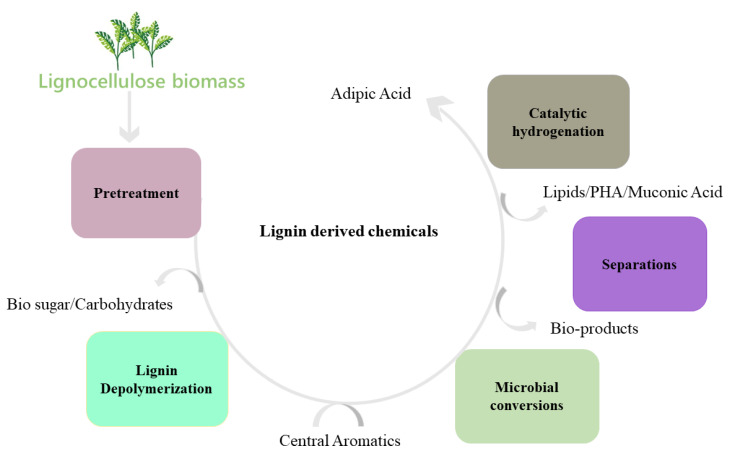
Important chemicals as byproducts from lignin degradation.

Multiple approaches have emerged for lignin valorization; the variation in these depolymerization methods and the original lignin source results in the formation of variety of products (Figure 5). The lignin isolation from lignocellulose will enable the removal of cellulose/hemicellulose by solubilization, leaving insoluble lignin residues or, in contrast, the removal of lignin and leaving insoluble residues of cellulose/hemicellulose [20,65]. The various types of depolymerization methods, including physical, chemical, physiochemical and biological methods, are reviewed in Table 3 below. 

## 5. Enzymatic and Biological Depolymerization

Due to the complex bond types and heterogeneous characteristics in lignin, it cannot be cleaved by hydrolytic enzymes like other natural polymers such as cellulose, starch and protein. However, enzymatic methods often involves a series of special non-specific fungal and bacterial lignin-degrading oxidoreductase enzymes or fenton’s reactions breaking a broad range of chemical linkages within lignin. The oxidoreductases, including laccase, manganese peroxidase (MnP), lignin peroxidase (LiP), versatile peroxidase (VP) and a unique dye-decolorizing peroxidase (DyP) are used in generating reactive radicals to destruct the lignin to a slate of reactive intermediates [15,20,100,116]. Laccase is known to have a low redox potential, which can act on phenolic structural units of lignin and non-phenolic structural units such as p-coumaric acid, 2,2′-azino-di (3-ethylbenzthiazoline-6-sulfonic acid) syringaldehyde and vanillin in the presence of mediators acting as electron shuttles. Previous studies have shown that the synergism of laccase improves the lignin action of microbial strains and increases its biomass, thereby enhancing lignin conversion [117,118,119,120]. LiP have a higher redox potential and attack non-phenolic lignin units by producing intermediate radicals [121]. MnP can chelate and oxidase Mn^2+^ to Mn^3+^, acting on both phenolic and non-phenolic lignin structural units, via the lipid peroxidation reaction, to depolymerize natural and synthetic lignin and entire lignocelluloses in vitro. This depolymerization effect could be enhanced by the presence of co-oxidants such as thiols or unsaturated fatty acids and their derivatives [17,122]. For enzymatic depolymerization, it is crucial to avoid the formation of inhibitors for microbial growth and to improve the reaction rate. In addition to the enzyme depolymerization of lignin, a series of free radical reactions mediated by Fenton’s reaction also plays an essential role in lignin depolymerization.

### 5.1. Microorganisms Involved in Lignin Depolymerization

#### 5.1.1. Fungi

With the rapid development of multi-omics technologies, more and more microorganisms have unveiled their capability to convert lignin into fungible fuels and products [123]. Fungi, including most white-rot and some brown-rot, have been widely applied in lignin deconstruction and the remediation of structurally similar pollutants for many years [124,125]. Several studies have reported that some fungi are responsible for releasing several valuable phenolic precursors, such as syringyl alcohol, ferulic acid, vanillic acid and protocatechuic acid from lignocellulose biomass, but most of these high-value compounds are hardly captured into intermediates and thus are not capable of large-scale fermentation [126,127]. Some of the important fungi efficient in lignin degradation are given in Table 4.

#### 5.1.2. Bacteria

**Figure 6:**
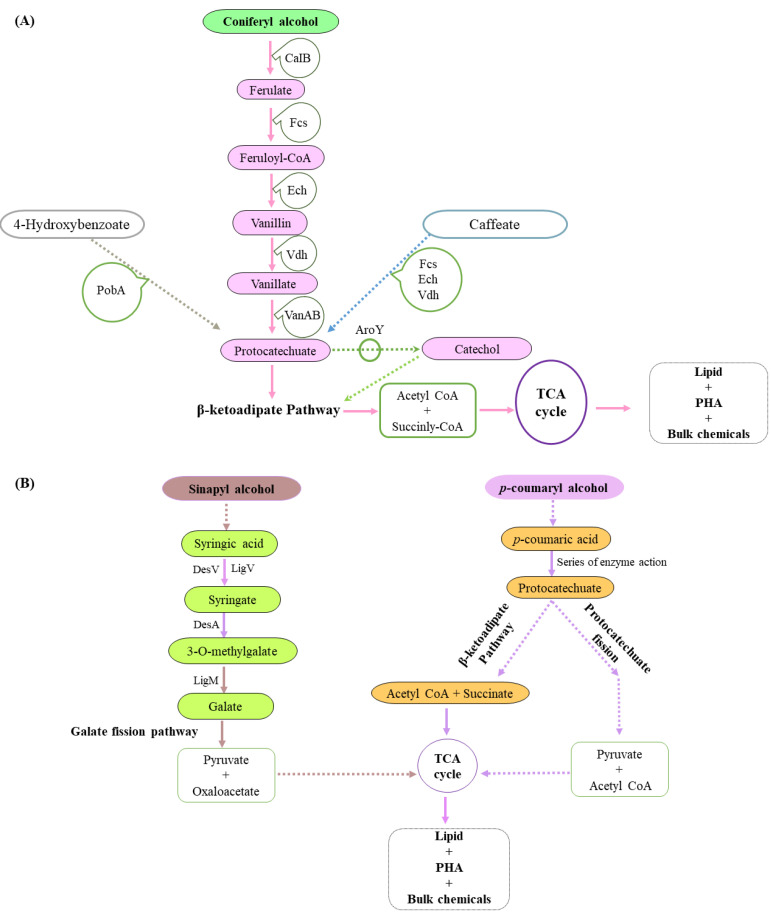
Aromatics catabolism from the coniferyl, sinapyl and p-coumaryl branch. (**A**) Conversion of diverse compounds like the coniferyl-alcohol, 4-hydroxybenzoate and caffeate to aromatics protocatechuate and catechol occurs, which in turn are processed through the different enzyme systems involved in the β oxidation pathway. The acetyl CoA and succinyl CoA formed then goes through the TCA cycle, leading to the formation of triglycerols lipids, PHA, and other fine chemicals. (**B**) The sinapyl alcohol forms pyruvate and oxaloacetate through the Galate fission pathway, which enters to TCA cycle, leading to the formation of fine chemicals. In contrast, the p-coumaryl alcohol forms succinate + acetyl CoA through the β-ketoadipate pathway and pyruvate + acetyl CoA through the protocatechuate fission pathway, which in turn enter the TCA cycle and help in the formation of lipids, PHA or other bulk chemicals.

Apart from fungi, the extent of lignin degradation by bacteria is not quite as extensive. However, some well-known lignin degraders, such as *Actinobacteria*, α-*Proteobacteria*, and γ-*Proteobacteria*, collect and transform diverse compounds like coniferyl-alcohol and vanillate to intermediates such as catechol and protocatechuate via peripheral pathways [107,140,141]. These bacteria are capable of secreting enzymes for deposing different origins of lignin or lignin-derived compounds and converting them to precursors for bioproducts. The end-products, such as succinyl-CoA and acetyl-CoA obtained via the β-ketoadipate (β-KAP) pathway, are further converted through the central carbon metabolism to produce polyhydroxyalkanoate (PHA), lipids and other chemicals, as shown in Figure 6. Therefore, these strategies offer a direct and versatile means to funnel the heterogeneous collection of molecules produced from lignin depolymerization to targeted intermediates. The intermediates then form fuels, chemicals, and other materials via a “biological funneling” approach [142,143]. 

Certain well-studied bacterial strains, including *Cupriavidus Necator* H16 and *Pseudomonas.* *putida* KT2440, are reported to be excellent candidates for converting lignin-derived aromatic compounds to polyhydroxyalkanoates (PHA) in lignin-enriched biorefinery streams and can even accumulate significant amounts of muconate from lignin-derived aromatics [144,145,146]. Similarly, *R. jostii* RHA1 and some other *Rhodococcus* species such as *R. opacus* DSM1069 and *R. opacus* PD630 have also exhibited their ability to accumulate triglyceride lipids by converting different lignin sources [142,147,148]. A few recently isolated bacteria such as *Pandoraea* sp. B-6, some other bacillus species, and a fresh discovery from a thermophilic environment have also shown their potential to convert lignin into a high-value product [149,150,151]. Most of the important lignin-degrading bacteria responsible for the bioconversion of lignin to valuable products like lipids, PHA, and mucanoic acid are shown in Table 5.

### 5.2. Metabolic Pathways Involved

Numerous catabolic pathways have been reported to be involved in lignin degradation, including some renowned pathways reviewed by [164]. The biphenyl and β-aryl ether (β-O-4) catabolic pathways are of significant importance, in the sense that both these pathways are highly dominant in lignin degradation. Most importantly, certain value-added chemicals, including vanillin and 4-hydroxybenzoic acid, can be created from lignin through such pathways [165]. Correspondingly, the β-ketoadipate (β-KAP) pathway likewise plays a prominent role in lignin bioconversion; as lignin-derived aromatics can be assembled into high-molecular-weight compounds, mainly polyhydroxyalkanoate (PHA) and lipids, via this pathway [166,167], as shown in Figure 6. This pathway has been discovered and described in numerous prokaryotes, such as Gram-negative bacteria belonging to *Sphingomonas paucimobilis* SYK-6, *Pseudomonas putida*, *Pseudomonas acidovorans*, and *Thermophilic geobacillus* and Gram-positive bacteria belonging to *Corynebacterium glutamicum*, *Streptomyces viridosporus*, *Rhodococcus opacus,* and *Rhodococcus josti*. The exact pathway has also been reported in eukaryotes, including the rot fungi *Phanerochaete chrysosporium* and *Trametes versicolor* and the filamentous fungi *Aspergillus* sp., as well as in unicellular yeasts [106,107,164,166,167,168].

Although there is a great diversity in the catabolism of lignin and lignin-derived aromatic compounds by different prokaryotic and eukaryotic microorganisms, all current studies indicated two nodal products (catechol or protocatechuate), that are usually formed in the process of aromatic ring breakdown. This node is typically followed by aromatic ring fission and enzymatic conversion to central metabolites like acetyl-CoA and other constituents of the tricarboxylic acid (TCA) cycle [13,169,170].

## 6. Challenges and Strategies for Lignin Valorization

Even though some progress has been made in lignin valorization, effectively harnessing the intrinsic capabilities of biology to valorize lignin will require substantial research and developmental efforts. Some of the challenges that are of primary concerns and that need to be considered during the lignin valorization process and the possible strategic solutions to enhance the microbial biotransformation of lignin are discussed below. 

### 6.1. Selection of Lignin Source and Depolymerization Capacity

The first and most challenging step is to depolymerize lignin to monomeric or dimeric/oligomeric compounds for further utilization by microorganisms. Recent literature has reported that bacteria can depolymerize and convert renewable lignin from different sources. However, the depolymerization and utilization efficiency is low due to the lignin macromolecule’s severe recalcitrance. Even though some bacteria can use lignin-derived aromatic compounds, the lignin-depolymerization capacity of most of these bacteria is relatively weak compared to white-rot fungus [62,142,171]. For achieving high product titer and efficient lignin utilization, it is critical to conduct efficient lignin-depolymerization that could degrade heteropolymers into aromatic compounds [172,173]. Lignin sources affect lignin components in the process of depolymerization, so adjusting the source of lignin and lignin manipulation is the first strategy for promoting the efficiency of lignin depolymerization. Previous research indicated that the efficiency of lignin depolymerization could be significantly affected by the origins and types of lignin used. Improvements could be made to approve the associated relationship between lignin characteristic features and their conversion yield, as well as the final production efficiency [174,175]. 

### 6.2. Selection of Ideal Microorganisms

Depolymerization approaches will result in many heterogeneous aromatic compounds. Many of these products are likely to be detrimental to the growth of bacteria, such as phenol, vanillin, 4-hydroxybenzoic acid, and coumaric acid [176,177,178]. One of the challenges is reducing or avoiding the influence of lignin-derived phenolic monomers and tannin derivatives on microbial growth. To address this challenge, it is important to screen and select appropriate microorganisms for utilizing and tolerating a broad range of lignin-derived aromatic compounds. The ideal microorganisms need not only be capable of utilizing these heterogeneous compounds but also should adapt to the potentially unfavorable compounds derived from lignin depolymerization and must be domesticated for use in industrial bioreactors [179]. 

*Oceanimonas doudoroffii* was recognized as a functional strain that was isolated from a contaminated marine environment and revealed the capacity of consuming lignin and its byproducts as the solitary carbon source for the synthesis of PHA [159]. Similarly, another vigorous bacterium, *Cupriavidus basilensis* B-8, screened from the steeping fluid of the erosive bamboo slips has been reported for its high capacity for lignin-derived aromatic compound catabolism [179].

### 6.3. Enzyme–Microbe and Microbe–Microbe Synergy 

Commonly, depolymerizing lignin into solely monomeric or dimeric aromatic compounds is challenging. Despite the unveiling of fungal and bacterial enzymatic machinery for lignin depolymerization, the genetic manipulation to engineer lignin bioprocess streams is still not much developed. Hence, the efficient depolymerization and conversion of lignin always requires the synergistic effects of different enzymes [180]. A recent report demonstrated that combining the commercial laccase from *Trametes versicolor* with an oleaginous bacterium, *Rhodococcus opacus* PD630, can selectively degrade different chemical linkages to synergize lignin depolymerization. Thus, the development of a simultaneous depolymerization and fermentation process results in the fast growth of cells and higher lipid yields on kraft lignin [181]. Some other work has reported that MnP can accelerate lignin depolymerization when applied in the anaerobic digestion of municipal solid waste [182]. Besides laccase and MnP, some fungi could produce a hydroxyl radical via Fenton’s reaction; thus, the lignin structure will be more accessible for the lignin-degrading enzymes through this chemical oxidation generated by Fenton’s reaction [183]. Meanwhile, some other lignin-degradation enzymes, such as lignin peroxidase, versatile peroxidase and the novel dye-decolorizing peroxidase, could be explored for further potential development to synergize lignin degradation. Another significant provision of lignin-degrading enzymes is engineering microorganisms to secrete the main heterologous enzymes in the target microbe to enable efficient lignin depolymerization. 

A biological team reported up to 20% lignin decomposition from some basidiomycetes, including *Coriolus versicolor*, *Trametes gallica*, lignin-degrading *Microbacterium* sp., and *Streptomyces* sp. for the generation of valuable chemicals due to potential synergy [181,184]. Similarly, lignin-degrading enzymes like laccase and MnP have been successfully expressed in *Escherichia coli* and yeast. However, the value of ligninolytic enzymes and its optimal mixture to degrade lignin synergistically for the synthesis of value-added products is currently unclear [15,185]. Therefore, advanced study is required to gain knowledge of potential interaction, which will help in the development of a lignin-degrading enzymes cocktail for lignin conversion to useful target products. Moreover, the application of biological omics-based techniques and advanced chemical analytics will not only provide an efficient tool to search for new enzymes but also new clues about how natural systems utilize these complex substrates. 

### 6.4. Optimization of Catabolic Pathways and Yield of Desire Product

The second essential and challenging step is to catabolize lignin-derived aromatic compounds into other value-added intermediates by microorganisms. Numerous researchers have proven the possibility of the biological valorization of lignin; however, a common problem is still the low yield of the final chemical products [106,186]. Despite lignin-degraders being continuously explored, there is still a lack of particular traits responsible for producing target products from lignin at a predicted level. Thus, the mechanism of their catabolic pathways suggests a possible approach to tackle the low yield of target products [107]. To obtain a higher yield of natural intermediates in the upper pathways of lignin catabolism, one effort could be made to block the advance metabolism of a desire product [187]. Several studies have unveiled opportunities for optimizing the yield of the final product via microbial pathway engineering; however, research on aromatic catabolism is solely concentrated on previously reported strains, such as *P. putida* KT2240, *R. jostii* RHA1 and *Cupriavidus Necator* H16. Therefore, it is necessary to explore those microorganisms that are efficient t changing lignin into value-added products and that eventually may reveal new pathways to deliver novel value-added products. Finally, a fundamental understanding of the lignin conversion process could optimize the conversion process of lignin derived from biorefinery into value-added products.

## 7. Conclusions and Outlook 

Lignin depolymerization is a hot topic of research due to its cost effectiveness and due to it acting as a renewable energy source for value-added biochemical synthesis. Therefore, the current study has reviewed the depolymerization and bioconversion of lignin to pave a way towards the synthesis of green and sustainable bioproducts. Preliminary investigations showed that nature includes many bio catalytic processes that can act synergistically with the various physical and chemical approaches to isolate and utilize the challenging lignin from lignocellulose biomass and convert it into energy-storage materials. Overall, the studies point to the fantastic potential and feasibility of lignin conversion into valuable bioproducts using catalytic approaches. However, fundamental understanding and extra studies are required to give insights into the potential interactions for adapting lignin-degrading enzyme cocktails. Moreover, further research on lignin pretreatments and catabolic pathway manipulation to develop an efficient bioconversion system is crucial for solving all of the bottlenecks in the process.

## Figures and Tables

**Table 1:** Different types of lignin, their monomer’s molecular weight, lignin content and chemicals.

	Types of Lignin	Source	Monomer Molecular Weight (g/mol)	Lignin Content (mmol g^−1^)	Chemicals/Catalysts	Reference
**Sulfur process**	Kraft lignin	Wood chips, softwoods, hardwoods	2000–3000	1.25	NaOH, Na_2_S	[34]
	Lignosulfonates	Softwoods, hardwoods, annual plants	20,000–50,000	1.25–2.5	Ca(HSO_3_)_2_ or Mg(HSO_3_)_2_	[34]
**Sulfur free process**	Organosolv lignin	Hardwood, softwood and wheat straw	2000–5000	0	Methanol, ethanol, various bronsted acid catalysts (H_2_SO_4_)	[28,35]
	Alkali/soda lignin	Hardwood, bagasse, wheat straw and flax	5000–6000	0	NaOH, NH_4_OH, Ca(OH)_2_	[36,37]

**Table 2:** Comparison of various lignin isolation methods.

Method	Source	Extraction Process	Approximate Average Mw (g/mol)	Approximate Average Mn (g/mol)	Advantages	Challenges	References
Milled wood lignin (MWL)	Milled sample particles and wood chips from LCB, wheat straw, redwood, white fir,	Extraction with a neutral organic solvent	9880	3367	Requires mild conditions and room temperature	Relatively low yield and time-consuming	[38,39,40]
Cellulolytic enzyme lignin (CEL)	MWL residue, redwood, wheat straw, white fir	Cellulolytic enzyme hydrolysis before aqueous dioxane extraction	19,830	5967	Requires mild conditions, no impurity and less inhibitor formation	Low yield of soluble and fragmented lignin	[39,41,42]
Enzymatic mild acidolysis lignin (EMAL)	Milled wood, hardwood, softwood, wheat straw, redwood, white fir	Cleaving lignin–carbohydrate bonds with the combined effect of enzymatic and mild acid hydrolysis	45,530	7717	Comparatively higher yield than MWL and CEL, with low severity	High concentration of HCl may compromise the structure of the isolated lignin	[39,40,43]

**Table 3:** Different pretreatment methods and lignin-degrading strategies along with advantages and limitations.

Depolymerization Method	Pretreatment	Feedstock	Strategy	Sugar Yield (%)	Advantages	Limitations	References
**Physical**	Pyrolysis and Gasification	Wide variety of lignin sources, including agriculture residuals and dry impregnated lignin	Cellulose carbonation at high temperature	Up to 85% reduction of sugars	Simple and inexpensive, and can be used for the processing of a large variety of feedstocks	A higher temperature initiates the decomposition of the products with a reduced yield of bio-oil.	[66,67,68]
	Fragmentation (Milling, Rolling, Grinding)	Wheat straw and a variety of other feed stocks	Disintegration of lignocellulose	Up to 70% reduction of sugars	Reducing crystallinity and particle size with no inhibitory compounds production	High energy is required.	[69,70,71]
	Ultrasonication	Woodchips, sugarcane bagasse, sugar beet shreds	Breaking hydrogen bonds in the lignocellulose conformation	20–50% reduction of sugars	Facilitating the disruption of several lignocellulosic materials	Prolonged sonication may cause an adversarial effect due to collisions between the particles.	[72,73,74]
	Microwave irradiation	Kraft pulp, hardwood, sawdust, sugarcane leaf wastes, wheat bran, rye bran barley husk and oat husk	Weakening the cellulose crystal structure	30–60% reduction of sugars	Operating easily and the ability to process bulky biomass with less inhibitor formation	High temperature and electricity consumption	[74,75,76]
	Extrusion	Wheat straw, sugarcane bagasse, deep litter, and sweet sorghum bagasse	Lignocellulose decomposition	50–75% reduction of sugars	Safe and the production of a significant amount of biogas	High energy consumption and the partial destruction of the lignin–carbohydrate complex	[77,78]
**Chemical**	Alkaline pretreatment	Agriculture residuals: sunflower stalk, wheat straw, rice straw, and corn stover	Modification of lignin through lignocellulose saponification	65–85% reduction of sugars	Requiring room temperature to operate	Inhibitor formation and needs expensive catalysts	[36,79]
	Acid hydrolysis (Dilute)	Agriculture residuals: wheat straw	Hemicellulose decomposition with lignin dissolution	45–80% reduction of sugars	No need for acid recycling, and providing a high glucose yield	Needs high pressure and temperature, while the formation of inhibitors also occur	[80,81,82]
	Acid hydrolysis (Concentrated)	Agriculture residuals: bagasse and wheat straw	Dissolution of hemicellulose and lignin	60–90% reduction of sugars	Operates at a mild temperature	Require corrosion-resistant equipment and needs expensive acids recovery	[83,84,85]
	Oxidation and ozonolysis	Agriculture residuals: bagasse, peanut, wheat straw, and poplar sawdust	Lignocellulose dissolution and the isolation of cellulosic crystals	45–90% reduction of sugars	Effective lignin degradation	Expensive ozone requirement	[86,87,88]
	Ionic liquids (ILs)	Agriculture residuals: bagasse, peanut, wheat straw, and corn stover	Breaking of hemicellulose bonds and cellulose sequestration from lignocellulose	60–85% reduction of sugars	Sufficient dissolution of cellulose	Exclusive amount of expensive ILs are needed.	[89,90,91]
	Organosolv	Agriculture residuals: sugarcane bagasse and wheat straw	Hydrolyzing hemicellulose with lignin removal	Reduction of sugars up to 60%	Comparatively low inhibitor formation	The removal of solvents before fermentation is costly.	[90,92,93,94]
	Deep eutectic solvent (DES)	Variety of lignocellulosic materials, including agriculture residuals and corncob lignocellulose	Biomass dissolution delignification and reducing cellulose crystallinity	Reduction of sugars up to 76%	Inexpensive, easy to prepare, highly tunable, and less toxic	Requires high temperature and combinatorial pretreatment, and sometimes produces unwanted impurities and increased viscosity	[95,96,97]
**Biological**	Enzymatic treatment	Agriculture residuals: rice straw, wheat straw, and softwood	Cellulose decomposition	20–50% reduction of sugars	Practiced under moderate conditions, and minimal energy is required.	Low hydrolysis rate, more time and a wide sterile area is required. Low hydrolysis rate and a wide sterile area is required.	[98,99,100]
	Fungal treatment	Agriculture residuals: rice straw, wheat straw, and softwood	Lignin and hemicellulose decomposition	20–50% reduction of sugars	Cost-effective, and moderate conditions and minimal energy is required.		[101,102,103]
	Bacterial treatment	Agriculture residuals: rice straw, wheat straw, and softwood	Hemicellulose and lignin decomposition	20–50% reduction of sugars	Cost-effective and requires moderate reaction conditions		[104,105,106,107]
**Physicochemical**	Steam explosion	Hardwood, forest residuals, sugarcane bagasse, wheat straw, and corn stover	Biomass delignification, hemicellulose solubilization and lignin transformation	50–70% reduction of sugars	Economical and safe	Inhibitor formation and the partial destruction of the lignin–carbohydrate complex	[108,109]
	Supercritical CO_2_ explosion	Sugarcane bagasse and wheat straw	Lignocellulose decomposition	Reduction of sugars up to 90%	No formation of inhibitory compounds	Non-economical and requires high pressure	[49,110]
	Liquid hot water (LHW)	Agriculture residuals: sugarcane bagasse, sunflower stalks, wheat straw, and corn stover	Hemicellulose hydrolysis to oligomers and acids	80–94% reduction of sugars	Minimum inhibitor formation, and no catalyst or any other chemical is required	High energy demand and excessive solid-mass generation	[111,112]
	Ammonia fiber explosion (AFEX)	Agriculture residuals: rice straw, wheat straw, corn stover, and sugarcane bagasse	Hydrolyzing hemicellulose with lignin removal, and the disruption of lignin–carbohydrate linkages	80–90% reduction of sugars	Operates under mild conditions, with minimum inhibitor formation	Expensive	[113,114,115]

**Table 4:** Lignin depolymerization through fungi.

Fungal Strains	Substrate	Strategy/Pathway	Enzymes	References
*Phanerochaete chrysosporium*	Synthetic lignin and free-hydroxyl phenolic groups	Multi enzyme approach, ortho-cleavage pathway, and phenanthrene metabolism	Lignin peroxidase, manganese peroxidase, dehydrogenase, engineered 4-O-methyltransferase	[128,129]
*Phanerochaete chrysosporium* *Irpex lacteus* CD2	Alkali lignin	The synergistic approach of the fungal co-culture	Nonspecific lignin-degrading enzymes	[130]
*Physisporinus vitreus*	Monomeric and dimeric, phenolic and nonphenolic lignin model compounds	Enzymatic hydrolysis of corn stover in vitro	Versatile peroxidase	[116,131]
*Ceriporiopsis* *subvermispora*	Nonphenolic lignin model monomers and dimers	One-oxidation-electron mechanism	Unidentified	[132,133]
*Ceriporiopsis subvermispora,* *Pleurotus eryngii* *Lentinula edodes*	Wheat straw lignin and structural motifs	Selective delignification	Unidentified	[134]
*Ceriporiopsis* *subvermispora*	Specificities for typical LiP/VP substrates	Lignocellulose de lignification through high selectivity	Newly discovered peroxidases	[135]
*Pleurotus ostreatus*	Lignin model dimer and synthetic lignin Corn stover lignin	Heterologous expression in *E. coli*, and eterologous expression in *Pichia pastoris*	Versatile peroxidases, manganese peroxidases, and aryl-alcohol dehydrogenase laccase	[136] [133]
*Trametes versicolor*	Phenol, *p*-creosol	Eukaryotic β-ketoadipate pathway	Laccases	[137,138]
*Anthracophyllum discolor*	Pyrene, phenanthrene, fluoranthene, anthracene, and benzo pyrene	Polycyclic aromatic hydrocarbon degradation in Kirk medium	Mn peroxidase; laccase and lignin peroxidase	[139]

**Table 5:** Lignin depolymerization through bacteria.

Strain	Substrate	Product	Strategy/Pathway	Enzymes	References
*Rhodococcus rhodochrous*	4-hydroxybenzoic acid, vanillic acid and glucose as the co-substrates	Lipid	Uses lignin model monomer	Enzymes involved in aromatic degradation and lipid accumulation	[152]
*R. opacus* PD630 *R. jostii* RHA1 VanA	Kraft lignin	Lipid	β-ketoadipate pathway and phenylacetic acid pathway, the co-culture of *R. jostii* RHA1 and *R. opacus*	Multiple peroxidases with accessory oxidases	[153]
*R. opacus* DSM 1069	O_2_ pretreated kraft lignin	Lipid	β-ketoadipate pathway, O_2_-based lignin pretreatment	Peroxidases and lipid biosynthetic enzymes	[154]
*Pandoraea* sp. ISTKB	Kraft lignin	PHA	CoA-mediated degradation pathways of phenylacetate and benzoate	Peroxidase-accessory enzyme system	[155]
Engineered *P. putida* KT2440 *Ralstonia Eutropha*. H16	Lignin Alkaline pretreated liquor	PHA PHA	The knocking out of *phaZ* and the overexpression of *alkK, phaG, phaC1,* and *phaC2* genes Two-step enzymatic hydrolysis	Multiple enzyme systems for PHA biosynthesis PHB biosynthetic pathway	[156] [157]
*Cupriavidus basilensis* B-8	Lignin	PHA	Phenol degradation pathways, β-ketoadipate pathway and the Gentisate pathway	Manganese peroxidase (MnP) and laccase	[158]
*Oceanimonas doudoroffii*	Lignin	PHA	Direct microbial conversion from lignin to biopolyester	Pathway not identified	[159]
*Corynebacterium. glutamicum* MA-2	Catechol	*cis, cis*-MA	Overexpression of *catA* gene and the deletion of *catB* gene	Catechol 1,2-dioxygenase	[160]
*Amycolatopsis* sp. ATCC 39116	Softwood lignin hydrolysate mainly guaiacol	*cis, cis*-MA	β-ketoadipate pathway, uses a metabolically engineered cell factory	Enzymes involved in the ketoadipate pathway, catechol dioxygenase and β-glucuronidase	[161]
*Pseudomonas. putida* KT2440	Alkaline pretreated liquor, *p*-coumaric acid	*cis, cis-* Muconate, Miconic acid	Integration of the *aroY* gene and the deletion of the *catB* and *catC* genes, controlling carbon catabolite repression	CatA and CatA2 dioxygenases	[162]
*Sphingobium* sp. SYK-6, *Pseudomonas. putida* KT2440	Hardwood lignin hydrolysate	*cis, cis*-MA	Using G-lignin components for *cis, cis*-MA production	Multiple enzyme system for *cis, cis*-MA production	[163]

## Data Availability

Not applicable.
